# Towards Predictive Computational Models of Oncolytic Virus Therapy: Basis for Experimental Validation and Model Selection

**DOI:** 10.1371/journal.pone.0004271

**Published:** 2009-01-30

**Authors:** Dominik Wodarz, Natalia Komarova

**Affiliations:** 1 Department of Ecology and Evolution, University of California Irvine, Irvine, California, United States of America; 2 Department of Mathematics, University of California Irvine, Irvine, California, United States of America; Center for Genomic Regulation, Spain

## Abstract

Oncolytic viruses are viruses that specifically infect cancer cells and kill them, while leaving healthy cells largely intact. Their ability to spread through the tumor makes them an attractive therapy approach. While promising results have been observed in clinical trials, solid success remains elusive since we lack understanding of the basic principles that govern the dynamical interactions between the virus and the cancer. In this respect, computational models can help experimental research at optimizing treatment regimes. Although preliminary mathematical work has been performed, this suffers from the fact that individual models are largely arbitrary and based on biologically uncertain assumptions. Here, we present a general framework to study the dynamics of oncolytic viruses that is independent of uncertain and arbitrary mathematical formulations. We find two categories of dynamics, depending on the assumptions about spatial constraints that govern that spread of the virus from cell to cell. If infected cells are mixed among uninfected cells, there exists a viral replication rate threshold beyond which tumor control is the only outcome. On the other hand, if infected cells are clustered together (e.g. in a solid tumor), then we observe more complicated dynamics in which the outcome of therapy might go either way, depending on the initial number of cells and viruses. We fit our models to previously published experimental data and discuss aspects of model validation, selection, and experimental design. This framework can be used as a basis for model selection and validation in the context of future, more detailed experimental studies. It can further serve as the basis for future, more complex models that take into account other clinically relevant factors such as immune responses.

## Introduction

Oncolytic viruses are live replicating viruses that selectively infect cancer cells and kill them [1]–[14]. Healthy cells are largely spared. The idea is to inoculate the virus into a cancer patient, and let the virus spread throughout the tumor, thereby driving it into remission. Selectivity for cancer cells occurs because cancer cells tend to lack important genes that normally shut down the replication cycle of the virus. For example, the adenovirus ONYX-015 has been engineered such that it only replicates in p53−/− cells, a characteristic of many cancers [10]. Certain animal viruses by chance have the ability to replicate in human cancer cells, while healthy human cells are not permissive. An example is Newcastle disease virus, which can replicate in tumor cells that lack interferons [7], [15]. In general, a wide array of viruses is being explored as potential oncolytic viruses.

Oncolytic viruses have shown promising results in clinical trials [16]. Cancers have been found to respond to treatment, leading to tumor remission in some cases. Consistent and sustained eradication or control of cancers has, however, been very difficult to achieve. This is caused in part by our lack of understanding regarding the dynamics that underlie the spread of oncolytic viruses through tumors. Without such a rigorous understanding, much of the work is based on trial and error. In such scenarios, mathematical models can be very useful to complement empirical work. Mathematical analysis allows us to see the whole spectrum of possible outcomes, and provides a means to logically suggest ways to optimize treatment. Limited mathematical analysis of oncolytic virus therapy has been performed in the past [17]–[21]. This work is largely qualitative in nature, examining how variation in viral and host parameters influences the outcome of treatment. For example, it has been suggested that maximizing the virus-induced rate of tumor cell killing is not going to lead to the best treatment outcomes. Instead, an intermediate and optimal rate of virus-induced cell death optimizes treatment success [17], [18]. This work was based on the analysis of the equilibrium properties of the model. That is, the lower the total number of cancer cells that remain as the dynamics converge to steady state, the better the predicted outcome of therapy.

While such steady state analysis can provide some valuable qualitative insights, it has limitations. The main problem is that in such infection dynamics models, the population of cells and viruses can show extensive oscillations before converging to a steady state. During these oscillations, the populations of cells and viruses can potentially go extinct, and the system might never reach equilibrium. Therefore, it is important to understand these oscillatory dynamics, and how they relate to the chances that the cancer cell population is driven extinct.

This paper aims to analyze these dynamics in detail in an attempt to provide a more realistic description of oncolytic virus dynamics. This is a difficult task because these infection dynamics, and in particular the occurrence of population oscillations, can be dependent on particular details of the models that are of a biologically uncertain nature. To address this issue, we avoid concentrating on a particular model, but take a more general approach. Through specific restrictions about biological assumptions, we analyze a class of mathematical models that aim to describe viral spread through a tumor in different settings. We seek to determine conditions under which the virus is successful at eliminating the tumor, and the conditions when virus therapy fails. In order to underline the insights that we gain from this general framework, we also consider specific models that are examples of the general framework. This modeling framework provides the basis for experimental validation and testing procedures, which will allow us to accurately predict the time course of cells and viruses at least in relatively simple scenarios, such as in vitro experiments or simple in vivo scenarios. In this context, we fit the models to previously published experimental data and discuss implications for model testing, model selection, and experimental work. A predictive model of a complex in vivo situation (e.g. including immune responses) will obviously be more difficult to attain, but can arise from a thorough understanding of the simpler in vitro scenario that we examine here.

## Results

### The modeling framework

We will model the dynamics of oncolytic virus replication by ordinary differential equations that describe the development of the average population sizes of cells and viruses over time. This approach is based on very well established mathematical models that describe the general dynamics of virus spread both in vivo and on an epidemiological level [22], [23]. Instead of considering a specific model, however, we will take a generalized approach and consider a class of models. The general modeling framework used in our study is as follows. We take into account two populations: uninfected tumor cells, *x*; and infected tumor cells, *y*. The population of free viruses is not modeled explicitly. Because the turnover of free viruses is much faster than that of infected cells, we simply assume that the free virus population is in a quasi-steady state and proportional to the number of infected cells. The basic model is given as follows:
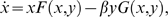
(1)

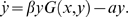
(2)


The function *F* describes the growth properties of the uninfected tumor cells, *x*, and the function *G* describes the rate at which tumor cells become infected by the virus. These functions are unknown and can potentially take a variety of forms, which will be discussed below. The coefficient *β* in front of the infection term represents the infectivity of the virus. Finally, virus-infected cells die with a rate *ay*. We will not include immune responses in our considerations. While immune responses will certainly be an important factor for oncolytic virus dynamics in vivo, our goal is to first understand those dynamics in a simpler setting without the presence of immune responses. These models would be suitable to describe the growth of oncolytic viruses in relatively simple in vitro or in vivo settings. Once an understanding of such simple systems has been achieved, additional biological complexities (such as the presence of immune responses) can be added to the model.

This class of models is characterized by the existence of equilibria, the number and nature of which depends on the tumor growth term *F* and the infection term *G*. In the most general sense, the equilibria of the system are defined by the following two equations:
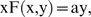
(3)

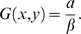
(4)We will explore the equilibria and their properties depending on the tumor growth term, *F*, and the infection term, *G*.

The term *F* reflects the growth properties of an uninfected tumor. It comprises both division and death rates. The simplest assumption that can be made about the term *F* is that growth is exponential (or, more precisely, the division and death happen according to an exponential law, and the division rate is higher than the death rate). While this can be true during early stages of tumor growth, tumor growth certainly deviates from an exponential pattern at larger sizes for a variety of reasons, for example space or nutrient limitations. Therefore, more complicated tumor growth terms involving some form of saturation must be considered [24]. In this respect, we can distinguish between two basic scenarios: First, while the rate of tumor growth saturates and slows down at higher tumor sizes, the tumor has the potential to keep growing towards infinity. Growth would stop once the tumor has reached a lethal size. Second, it can be assumed that growth not only slows down, but comes to a halt as the tumor size reaches a critical level, which can be called the carrying capacity of the tumor. This could happen when the division rate equals the death rate of the cells.

Regarding the infection term, the assumption used most often in mathematical models is that it is directly proportional to the number of infected and uninfected cells [25], [26]. This, however, assumes mass action or perfect mixing of populations, which is unrealistic, especially in the context of tumors. Instead, virus spread is likely to be slower, limited by spatial constraints. Since the virus released from one infected cell cannot reach all susceptible tumor cells in the population, the infection rate must be a saturating function of the number of susceptible tumor cells. Similarly, not all infected cells present in the population will be able to contribute to the generation of newly infected cells, for example if they are spatially separated from susceptible cells.

In the following section, we will define different classes of infection terms that have biologically reasonable characteristics, and investigate how they influence the properties of the model. These are based in part on mathematical work done in the context of infectious disease epidemiology [25], [26]. Subsequently, we will examine how changing the tumor growth term influences the model predictions.

### Different classes of infection terms and their properties

**Figure 1:**
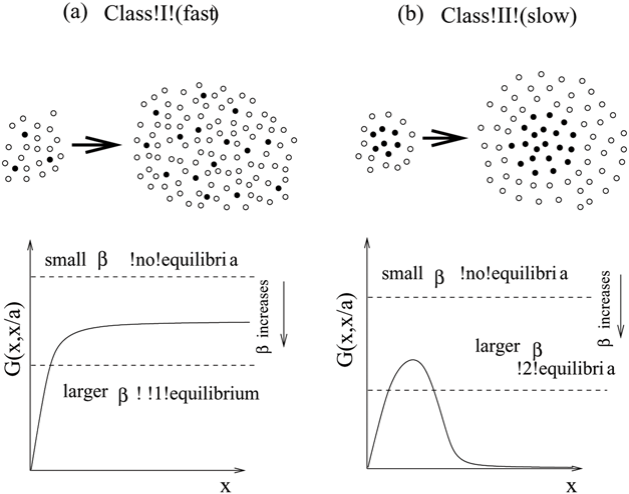
Two classes of virus growth captured by the mathematical models. (a) According to class I or fast virus growth, virus growth is exponential as long as the number of uninfected cells is significantly larger than the number of infected cells. This can correspond to a high degree of mixing between infected and uninfected cells. As the virus population grows, the number of cells that contribute to virus spread remains constant because most infected cells will have an uninfected cell in their vicinity. (b) According to class II or slow virus growth, virus growth slows down and saturates as the virus population increase in size, even if the number of uninfected cells is relatively large. This can correspond to spatial clustering of the infected cells. Only infected cells at the surface have uninfected cells in their neighborhood and can thus contribute to virus transmission. As the number of infected cells rises, the number of “active” cells that can contribute to virus transmission declines.

Let us consider two different classes of viral growth, see figure 1(a,b). Tumor-virus systems belonging to class I are characterized by the following property: if the number of uninfected tumor cells is high relative to the number of infected cells, virus growth does not slow down as the number of infected cells rises. Virus growth is exponential. Biologically, this can be interpreted as virus replication in a non-solid tumor where cells mix relatively freely. In other words, infected cells are not clustered together in a mass but are interspersed among uninfected cells. This is shown schematically at the top of figure 1(a) (the white circles represent uninfected cells, and the black circles - infected cells). In this case, if the number of uninfected cells is relatively large, then every infected cell is likely to be surrounded by uninfected cells to which the virus can be passed on. Alternatively, a similar picture can be achieved by a very high motility of the virus. In either case, all infected cells contribute to viral spread and growth is exponential. We call this “fast virus spread”. On the other hand, with tumor-virus systems that belong to class II, the virus growth rate decreases as the number of infected cells rises, even if the number of uninfected cells is very large. The biological interpretation is that infected tumor cells are clustered together, figure 1(b). This can occur in solid tumors, which typically show a high degree of spatial arrangement. In this case, as the number of infected tumor cells increases, most infected cells will be surrounded by other infected cells and not by uninfected cells. Hence, they cannot pass on the virus and cannot contribute to virus spread. Only cells at the periphery of the infected cell mass have uninfected cells in the neighborhood and can contribute to new infection events. We refer to this model of infection as “slow virus spread”.

Next let us connect this classification with the mathematical model, and in particular, with the infection term, *βyG(x,y)*. The function *G(x,y)* is related to the proportion of the total population of the infected cells which participates in the infection process. It is plotted in figure 1 as a function of the number of tumor cells, *x*, and we examine the shape of these plots. Let us take a closer look at the schematic at the top of figure 1(b). Because of the geometrical arrangement of the cells in this case, only the infected cells on the surface of the black core will be able to infect other cells (it is 6 out of the 7 cells in the smaller colony presented). Now, let us increase the system size, such that the number of infected and uninfected cells grows in the same proportion. Again, only the infected cells close to the surface of the infected core will participate in the infection process. However, now the proportion of the surface cells is much smaller (11 out of 20 cells). As the size of the system increases, the proportion of such “active” cells (that is, cells capable of infecting other cells) decreases. This is what is depicted in the graph in figure 1(b), where the function *G(x,y)* declines following the peak. (For very small system sizes, the proportion of cells participating in infection is formally zero because of the lack of uninfected cells, therefore the graph of the function *G* starts at zero, reaches a peak, and then declines for high values of *x*). Next, we take a look at the cell arrangement at the top of figure 1(a). Here, the populations are well-mixed, and as the system grows, a constant fraction of infected cells will be able to infect new cells. This is reflected in the corresponding graph of *G(x,x/a)*, which reaches an asymptote and does not decline. In Table 1 we list several examples of fast and slow growth laws.

**Table 1:** Examples of different virus spread terms, G(x,y).

*G(x,y)*	Law of virus spread
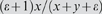	Fast (frequency dependent)
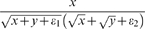	Fast
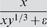	Slow
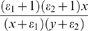	Slow
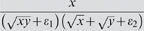	Slow
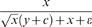	Slow

In general, we can prove that the two scenarios above are the only possible outcomes, given the biological requirements imposed on the function *G*. As *x* increases, this function increases, and can either approach zero or a nonzero level. If it approaches a non-zero level, this does not necessarily need to occur via a monotonic approach to the asymptote. It is possible that the function *G* first increases, peaks, and then converges to a non-zero asymptote. For intermediate values of *x* the function *G* may have a more complicated structure than that shown in figure 1, but in the absence of any biological evidence of that it is a safe bet to assume the simplest shape with a minimal number of local extrema.

How does the shape of *G* help us draw meaningful conclusions about the behavior of the biological system? It turns out that the function *G* is essential in determining the number and the stability properties of the equilibria of the system, and thus it will help us reason about long-term predictions on the treatment outcome.

Equations (3–4) can be combined in a single equation,
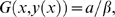
(5)where the function *y(x)* is a relationship between the number of infected and uninfected cells at equilibrium as the total system size grows; it is obtained from equation (3) and depends on the exact rate of cancer growth, *F*. If the cancer growth is exponential (*F = 1*), we have *y(x) = x/a*, that is, at equilibrium, the infected cells comprise a fixed fraction of uninfected cells. Thus the function *G(x,x/a)* depicted in figure 1 is just the left hand side of the equation for the equilibria, equation (5). The right hand side is represented by horizontal dashed lines, whose level decreases with the viral replication rate *β*. The number of intersections corresponds to the number of equilibria in the system.

We can see that the two graphs in figure 1 exhibit different numbers of equilibria. First we consider figure 1(a), fast virus spread. In this case, the model always contains a parameter region in which exactly one equilibrium exists. If the viral replication rate, *β*, lies below a threshold (*β*<*β_c_*) then no equilibrium exists. If the viral replication rate lies above that threshold, the following is observed. As shown in figure 1(a) exactly one equilibrium is found. In other cases, it is possible that there are two or more equilibria for intermediate viral replication rates. (For example, if the function *G(x,x/a)* first rises and achieves a maximum before descending to its horizontal asymptote, or if it goes through a number of local extrema before approaching a horizontal asymptote.) The most important universal feature in all fast growth scenarios is that for sufficiently high values of *β*, there is exactly one equilibrium. Next, consider Figure 1(b), slow virus spread. Again, for an equilibrium to exist, the viral replication rate needs to lie above the threshold *β*>*β_c_*. If this is the case, the system is always characterized by the presence of not one, but two equilibria. Again, in some cases, it is possible that the intermediate values of *β* correspond to more than two equilibria.

The biological interpretation of this analysis is as follows. We saw that for both modes of infection, if the values of the viral replication rate *β* are small, no equilibria exist. This translates into an uncontrolled cancer growth. This is an intuitive result: for low viral replication rates, treatment is impossible. A less intuitive result is connected with the number of equilibria once *β* is above its threshold value.

The cancer-virus system displays a fundamentally different behavior depending on whether it is characterized by one or two equilibria. If there is only one equilibrium, then the dynamics will be governed by the properties of this equilibrium only. Because the number of tumor cells is relatively low at this equilibrium, this outcome corresponds to containment of the tumor by the virus. For convenience, we call this internal equilibrium *E_I_*. On the other hand, the situation is more complicated if the system is characterized by two equilibria. The first equilibrium, at which the number of tumor cells is lower, is again the internal equilibrium, *E_I_*, and can be interpreted as containment of the tumor by the virus. The second equilibrium can be shown to be an unstable saddle node equilibrium, call it *E_S_*. The presence of the saddle equilibrium means that the dynamics are qualitatively different depending on the initial conditions. If the initial number of tumor cells is relatively low and close to the internal equilibrium, then the dynamics are governed by this internal equilibrium, *E_I_*, leading to a degree of tumor control. If the initial number of tumor cells is higher and around or above the saddle node equilibrium *E_S_*, then the number of tumor cells increases in an uncontrolled fashion. Hence, in this regime, uncontrolled cancer growth is always a possible outcome.

We conclude that our biologically defined modes of virus spread correspond to very different mathematical properties. Models of class I (fast virus spread) contain a parameter region (of high enough *β*) in which only a single equilibrium is observed. In this case, the model contains a parameter region in which uncontrolled cancer growth is impossible. Models of class II (slow spread) never have only one equilibrium and the saddle node equilibrium *E_S_* is present whenever the internal equilibrium *E_I_* exists. In this class of models, no matter how high *β* is, uncontrolled cancer growth is always a possibility.

### Effect of the tumor growth term

For the purposes of classification of the virus spread terms, we looked at the changes in *G* as the number of infected and uninfected cells grew in the same proportion. This led to a direct evaluation of the number of equilibria for exponential cancer growth (*F = 1*). While mathematically the simplest scenario, exponential growth is an unrealistic assumption, because the growth of cells is bound to saturate as the tumor grows. Our methods allow to study any reasonable cancer growth law in a very natural way.

Let us model a slow-down of the tumor growth rate as the number of tumor cells increases. This can be done in two different ways. On the one hand, we can assume that while tumor growth slows down, it never stops, such that the tumor can grow towards infinity over time. That is, there is no upper limit to the number of tumor cells; in practical terms growth will stop when the organism dies. An example is what we call “surface growth”, where only the cells around the surface of the tumor can give rise to viable daughter cells and can contribute to tumor spread. This can apply to solid tumors that have a high degree of spatial structure. Surface growth in 2D and 3D are listed in Table 2. The parameter *η* determines the tumor size at which saturation comes into play. Another possibility that falls into this category is that the rate of tumor growth becomes linear as the number of tumor cells increases. In this case, tumor growth is even slower; we refer to it as “linear growth”.

**Table 2:** Examples of different tumor growth terms, F(x+y).

**F(x+y)**	**Growth Law**
1	Exponential
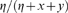	Linear
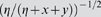	Surface growth in 2D
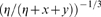	Surface growth in 3D
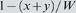	Logistic
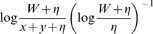	Gompertzian

On the other hand, it is possible that there is a natural limit or carrying capacity, *W*, that limits tumor growth [27]. Thus, we will assume that growth slows down and eventually stops as the number of tumor cells increases. This can occur in a variety of ways. Tumor growth can be exponential until the number of cells approaches carrying capacity and the rate of tumor cell growth becomes zero. For example, this can be described by the logistic growth term (see Table 2) [27]. Alternatively, we can assume that tumor growth first saturates according to the surface growth or linear growth patterns described above, and only reaches the carrying capacity once the tumor has grown to a significantly larger number of cells. Another example of a growth with a carrying capacity is a Gompertzian type growth [27], Table 2.

As mentioned before, the term *F* reflects implicitly both division and death properties of uninfected tumor cells. For example, an exponential growth is characterized by a net expansion rate resulting from exponential division and death processes. The logistic growth is a consequence of saturation of the division rate while the (exponential) death rate remains constant. In fact, any process with a sub-exponential division rate and an exponential death will be characterized by a finite carrying capacity. On the other hand, an unlimited (but saturated) growth (such as surface growth) implicitly includes death which happens slower than exponentially. If we were to add an exponential death term to a surface growth, it would lead to a limited growth with a carrying capacity. Our framework includes all these and any other reasonable functional forms of cellular growth.

In the following, we will examine the effect of different types of tumor growth terms on the properties of the model. We will do this first in the context of the faster virus infection terms that belong to class I, and then in the context of the slower infection terms that belong to class II. Note that our analysis is quite general and the particular growth laws listed in Table 2 are merely an illustration; the results are not restricted to these particular growth laws.

#### Effect on fast virus growth

With this class of virus infection term, we found that in the context of exponential tumor growth, *G(x,y(x))* with *y(x) = x/a* approaches a nonzero asymptote for large values of *x* (note that it can either rise monotonically to the asymptote, or first go through one or more local maxima before declining towards the asymptote). In either case, for any equilibrium to exist, the viral replication rate needs to lie above a threshold *β*>*β*_c_, and there exists a parameter region (characterized by values of *β* greater than a threshold) in which only the internal equilibrium *E_I_* is present. In this parameter region, tumor control is the only outcome.

Introducing saturated tumor growth (or changing the function *F* in any way) will lead to a different functional form of *y(x)* in equation (5). A universal feature is that any tumor growth slower than straight exponential growth will lead to smaller values of *y(x)* and thus to higher values of *G*. Therefore, as a result of tumor growth saturation, the asymptote becomes higher for slower tumor growth terms. This means that only the internal equilibrium *E_I_* can exist, as with exponential growth. The only difference lies in the viral replication rate threshold beyond which this equilibrium can exist and beyond which tumor control is possible. The slower the tumor growth, the lower the viral replication rate threshold required for virus-mediated control.

**Figure 2:**
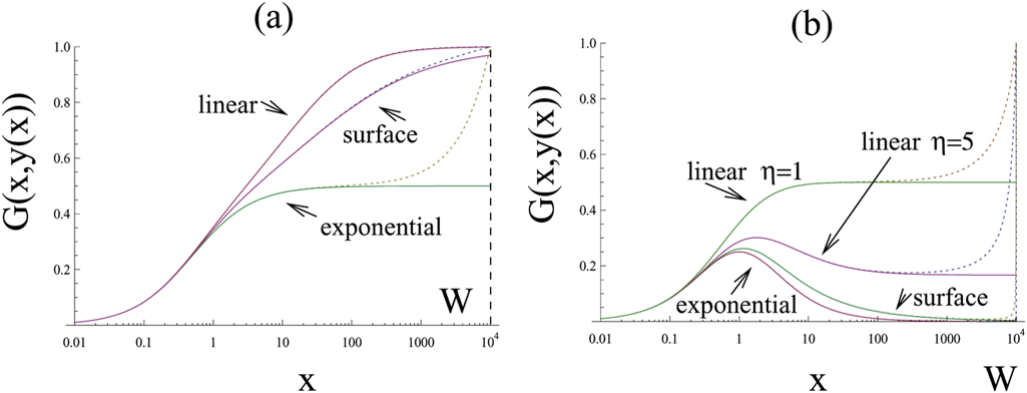
The effect of a carrying capacity. The function *G(x,y(x))* is plotted for two particular choices of the virus spread law and three different laws of cancer growth: exponential, surface growth and linear growth. (a) Fast virus spread, *G(x,y) = x/(x+y+1)* and (b) slow virus spread, *G = x/(x+1)/(y+1)*. The solid lines correspond to the unlimited cancer growth; the dotted lines - to a growth up to a given size, *W*. The parameters are: *a = 1*, *η = 10* and *W = 10^4^*.

If we assume saturated but limited tumor growth (i.e. growth stops at carrying capacity *W*), then the picture is similar for the most part, with one difference. After the term *G(x,y(x))* has approached the asymptote, the curve *G* takes an upward turn in the vicinity of *x = W*, i.e. when the number of cells approaches carrying capacity. This means that the model acquires an additional equilibrium, which corresponds to the cancer growing to its carrying capacity *W*. In the systems with unrestrictive growth, this was equivalent to unlimited growth of the cell population to infinitely large sizes. This is illustrated with the dotted line in Figure 2(a). We can see that for *x*≪*W*, the curves for limited and unlimited growth laws look identical, and near the carrying capacity *W* they deviate.

So far, we have concentrated on the case where *G(x,y(x))* increases monotonically towards an asymptote. Alternatively, the term *G(x,y(x))* can rise to a peak and then decline toward a non-zero asymptote. In this case, including saturation into the tumor growth term *F(x,y)* leads to similar consequences. However, the hump in the function can disappear, eliminating any parameter region in which both equilibria can exist. In other words, with slower tumor growth, there is no parameter region anymore in which the tumor can escape the effect of the virus and grow out of control. Whether this occurs or not depends on the relative size of the two spatial scales involved. The first scale is defined by the tumor size at which the virus infection function *G* saturates and peaks in the context of exponential growth; this is entirely dependent on the properties of the viral growth term. Let us call this scale *s_v_*, where the subscript refers to “viral”. The second scale is given by the colony size at which the tumor growth law starts to deviate from exponential; we will call this scale *s_t_* (where the subscript refers to “tumor”). When *x_t_*≤*s_v_*, the asymptotic value of *G* becomes sufficiently large such that the hump disappears. The disappearance of the hump makes treatment easier, and this occurs if tumor growth slows down before virus growth does.

#### Effect on slow virus growth

Here, we assume slower virus growth terms that belong to class II. In the context of exponential growth, the function *G(x,y(x))* first increases, and then declines towards zero. This means that if equilibria exist, both the internal equilibrium *E_I_* and the saddle node equilibrium *E_S_* are aways present. Consequently, the possibility always exists that the tumor can out-run the virus infection and grow uncontrolled. Taking into account saturated tumor growth has the following effect (Figure 2(b)). *(i)* The function *G* can remain qualitatively the same; that is, it rises to a peak and then declines towards zero. *(ii)* Alternatively, the picture can change such that it does not decline towards zero, but towards a non-zero asymptote, while remaining a one-humped function. *(iii)* Finally, the picture can change further such that the function *G* increases monotonically towards an asymptote. Which outcome is observed depends on the exact nature of the functions *F* and *G* and also the relative size of the two spatial scales involved: the tumor size at which the virus infection term *G* saturates and peaks (*s_v_*), and the size *s_t_* at which the pattern of tumor growth starts to deviate from exponential. Lowering the value of *s_t_* relative to *s_v_* shifts the outcome from scenario *(i)* to *(iii)*. As the value of *s_t_* becomes similar to the value of *s_v_*, the model contains parameter regions in which only the internal equilibrium *E_I_* exists and in which uncontrolled tumor growth is impossible. If s_t_≪s_v_, then the hump in the function *G* disappears, and the saddle node equilibrium *E_S_* is never present. In this case, virus-induced tumor control is the only outcome, and uncontrolled tumor growth cannot be observed. In biological terms, saturation of tumor growth at lower sizes promotes successful virus therapy. These arguments apply to all saturated tumor growth scenarios. With saturated but unlimited tumor growth, the function *G* approaches an asymptote for large tumor sizes *x*. For tumor growth that is limited by a carrying capacity *W*, the function *G* eventually deviates from the asymptote and rises again, indicating the presence of an equilibrium that describes tumor growth towards carrying capacity rather than towards infinity. Lowering the carrying capacity *W* has the same effect as lowering the parameter *s_t_* that determines the tumor size at which growth starts to saturate: it shifts the outcome from scenario *(i)* to *(iii)*.

### Summary of model properties

In summary this analysis has provided the following insights. We examined two types of infection terms and found that they strongly influence the dynamics of oncolytic virus spread. In the first class of models, virus spread was fast because infected cells are mixed among uninfected cells. In this case, tumor control is always observed if the viral replication rate lies above a threshold. In these parameter regions, loss of tumor control is not observed. In the second class of models, virus spread was assumed to be slow, because infected cells are clustered together in space. In this situation, the model can be characterized by bistability. If the initial number of tumor cells lies below a threshold, tumor control is observed. If the initial number of tumor cells lies above this threshold, uncontrolled tumor growth is observed. If tumor growth only saturates at high numbers of tumor cells or not at all, then uncontrolled tumor growth is always possible in parameter regions in which tumor control is possible. If tumor growth saturates at lower levels, there are parameter regions in which only the tumor control outcome is observed and in which uncontrolled tumor growth is not possible. If tumor growth saturates at even lower levels, then the bistability and the dependence on initial conditions vanishes completely.

### Properties of the internal equilibrium

The above analysis concentrated on the equilibria. By examining which equilibria exist under different conditions, we can obtain information about the ability of the virus to control the cancer, and about the possibility that the cancer grows despite the presence of the virus. If the dynamics are governed by the internal equilibrium *E_I_*, then the virus keeps the tumor cell population at relatively low levels and prevents uncontrolled tumor growth. We have discussed the conditions under which this can be achieved and interpreted these conditions from a biological angle. If the virus does control the tumor, however, additional questions arise. The virus can either control a persisting tumor at low levels, or the virus can drive the tumor cell population extinct.

Because we are considering ordinary differential equations that describe the average behavior of the cell and virus populations, true extinction cannot occur in this model. The number of cells can, however, drop to very low levels. If the average number of cells is below one, we can assume that tumor extinction is a likely event. Therefore, if the number of tumor cells at equilibrium lies below one, we can say that the virus is likely to drive the tumor extinct. However, even if the equilibrium number of cells lies above one, the tumor cell population can still go extinct during oscillatory dynamics that can occur before the dynamics reach equilibrium. Therefore, we need to understand the properties of the internal equilibrium in more detail. We will examine this in the context of both fast and slow virus growth. We will only assume saturated tumor growth and not consider straight exponential tumor growth.

#### Fast virus growth

One of the most important parameters that influence the properties of the internal equilibrium is the replication rate of the virus *β*. In general, the faster the replication rate of the virus, the lower is the equilibrium number of tumor cells. Further, it can be shown that if the viral replication rate *β* crosses a threshold, the behavior near the equilibrium becomes oscillatory. Both promote the eradication of the cancer. In general, the internal equilibrium can either be stable or unstable, depending on the particular model under consideration as well as parameter values.

**Figure 3:**
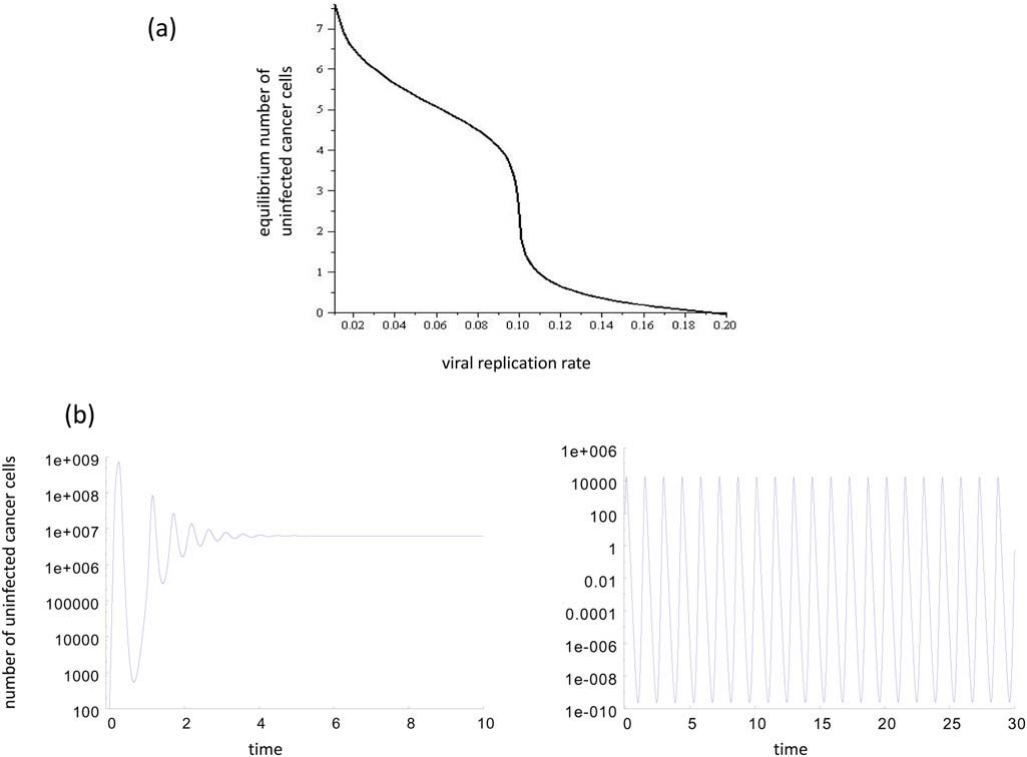
(a) The equilibrium number of uninfected cancer cells as a function of the viral replication rate *β* for fast virus growth. There is a threshold viral replication rate at which the number of cancer cells drops sharply from relatively high values to values of the order of one. This can be considered a tumor extinction threshold. (b) Dynamics of the uninfected cancer cells if the viral replication rate lies below (left) and above (right) this threshold. If the viral replication rate lies below the threshold, limited oscillations are observed that dampen out quickly. If the viral replication rate lies above the threshold, extensive oscillations are observed that reduce the cancer cell population to very low levels, and that dampen out very slowly (dampening not observed on time scale shown here). These plots were made by using a specific model from the fast virus growth category, that is *G = (ε+1) x/(x+y+ε)*. Note that the transition in oscillations is not a universal feature of all models in this class. Parameters were chosen as follows: *r_1_ = 1*; *a = 0.1*; *ε = 10*; *η = 10^8^*; *x_0_ = 100*; *y_0_ = 10*; For (b), *β = 0.07* and *β = 0.13*, respectively.

Let us first consider the case where the equilibrium is stable. Then we can distinguish between two parameter regions. Denote the size at which tumor growth slows down and deviates from exponential by *s_t_*. In the first parameter region, the value of *s_t_* is large compared to a value related to the virus scale, *s_v_* (for the exact definition see the Supporting information S1). In this parameter region, we observe a viral replication rate threshold, at which the equilibrium number of tumor cells drops sharply from relatively high values to values of the order *1* (figure 3a). This replication rate threshold can be defined for individual models that belong to this class and defines the condition for cancer eradication. If the tumor size at equilibrium drops to small values (of the order of *1* cell), stochastic effects are very likely to lead to extinction. This is further supported by changes in the oscillatory approach to the equilibrium, which we have investigated in the context of individual models (figure 3 b). At this viral replication rate threshold, the amplitude of the initial oscillations can increase sharply, as can the time it takes for the dynamics to approach the stable equilibrium (the real part of the eigenvalues of the Jacobian matrix rapidly approaches zero). Since pronounced oscillations reduce the number of tumor cells well below one, tumor eradication is the likely outcome. Note, however, that this drastic change in the oscillatory pattern is not observed in all models that belong to this class. The sharp drop in the equilibrium value is, however, a universal feature of models that belong to this class.

Now assume the other parameter region in which the scale *s_t_* is small. In this case, no such viral replication rate threshold exists. Instead, the equilibrium number of tumor cells declines proportional to the viral replication rate *β*. Numerical simulation of individual models, however, indicates that the minimum number of tumor cells can decline exponentially with an increase in the viral replication rate, although this could not be proved in general. Taken together, these findings indicate that in the parameter regions where virus replication is fast enough such that there is an oscillatory approach to the equilibrium, tumor eradication is the likely outcome.

As mentioned above, it is also possible that the internal equilibrium *E_I_* is unstable. In this case, we observe oscillations that diverge away from the equilibrium if the viral replication rate *β* is sufficiently fast. That is the amplitude of the oscillations increases over time. This is likely to correlate with extinction of the tumor, especially if the number of tumor cells at equilibrium is relatively low. This is because the oscillations will reduce the number of tumor cells well below the equilibrium value over time. Thus we conclude that for sufficiently large values of *β*, the cancer will be driven extinct by the virus through (convergent or divergent) oscillations.

#### Slow virus growth

In this case, the tumor size at the internal equilibrium is again negatively correlated with the viral replication rate *β*. Similarly to fast virus growth, the internal equilibrium can be stable or unstable depending on the individual model and on the parameter values. The dynamics will be discussed for both stable and unstable equilibria *E_I_*.

**Figure 4:**
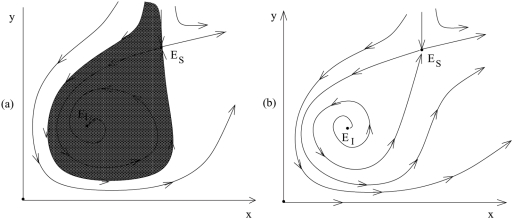
The phase portrait for a system with a slow virus propagation term. (a) The intermediate equilibrium, *E_I_*, is stable (the basin of attraction is shaded), (b) *E_I_* is unstable.

**Figure 5:**
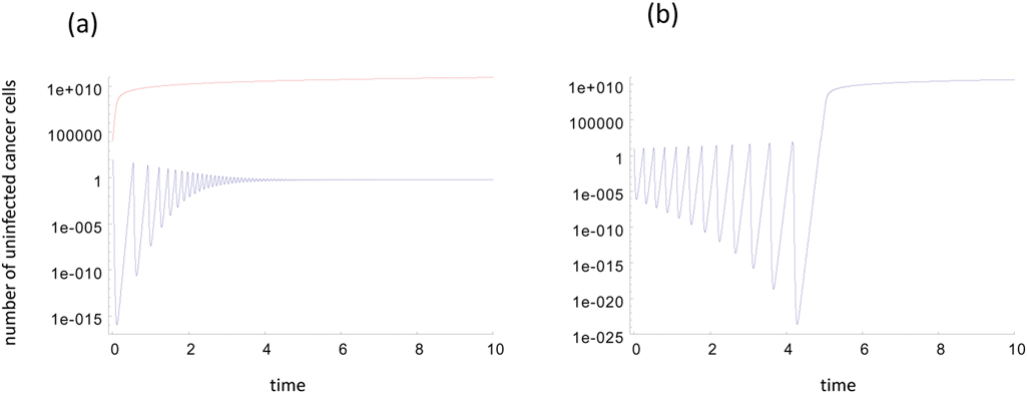
Dynamics in fast virus growth models assuming that the internal equilibrium *E_I_* is (a) stable and (b) unstable. (a) If the internal equilibrium is stable, then the dynamics can converge to this equilibrium via damped oscillation if the initial number of cancer cells is relatively low. On the other hand, if the initial number of cancer cells is relatively high, then uncontrolled cancer growth is observed. (b) If the internal equilibrium is unstable, then diverging oscillations are observed. Eventually, these diverging oscillations take the populations beyond the saddle node equilibrium, leading to unlimited cancer growth. Before that occurs, however, it is most likely that the cancer has been driven extinct in a stochastic setting because the diverging oscillations drive the tumor size to ever decreasing values. These plots were obtained from a specific model that belongs to the slow virus growth class, i.e. 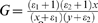. Parameters were chosen as follows: (a) *r = 1*; *β* = 0.8; *a = 0.5*; *ε_1_ = 20*; *ε_2_ = 10*; *η = 10^8^*; *x_0_ = 100* and *10,000*, respectively; *y_0_ = 10*. For (b) *r = 1*; *β = 1*; *a = 0.5*; *ε_1_ = 10*; *ε_2_ = 11*$; *η = 10^8^*; *x_0_ = 10*; *y_0_ = 1*.

If the equilibrium is stable, the approach is again oscillatory if the viral replication rate is sufficiently large (Figure 4a and 5a). Numerical simulations of individual models indicate that the minimum tumor size during these oscillations can decline exponentially with the viral replication rate *β*, although again this could not be proved in a general setting. These results indicate, however, that if oscillations are observed it is likely that the cancer is eradicated by the virus (Figure 5). Note that this assumes that the initial number of tumor cells is sufficiently small such that the population is in the region of attraction of the internal equilibrium. If this is not the case, the virus fails and unlimited virus growth occurs because the long-term outcome depends on the initial conditions as discussed above (Figure 5 a). In addition to these dynamics, the following can occur (Figure 4(a)). Assume that the tumor cell population is reduced to low levels during the initial oscillations, but not to extinction. As the tumor cell population rises again, it can actually cross over to values larger than the saddle equilibrium *E_S_*. Consequently, the cancer will grow uncontrolled and virus therapy will fail.

On the other hand, if the internal equilibrium is unstable, then the following is observed (Figure 4b and 5b). If the viral replication rate is fast enough, the populations show diverging oscillations away from the equilibrium (Figure 4b), i.e. the amplitude of the oscillations increases over time. During these diverging oscillations, the minimum number of tumor cells declines over time. Hence, the tumor is likely to hit extinction. Again, there is the possibility that during the oscillations the tumor cell population crosses over to values larger than the saddle equilibrium *E_S_*. In this case, the tumor cell population would grow uncontrolled to ever increasing levels.

To summarize, for slow virus growth oscillations around the internal equilibrium have the potential to drive the tumor cell population extinct. However, the bi-stability of this system causes problems since there is always the possibility that the populations can escape to large numbers, leading to uncontrolled tumor growth.

### Application of models to experimental data

**Figure 6:**
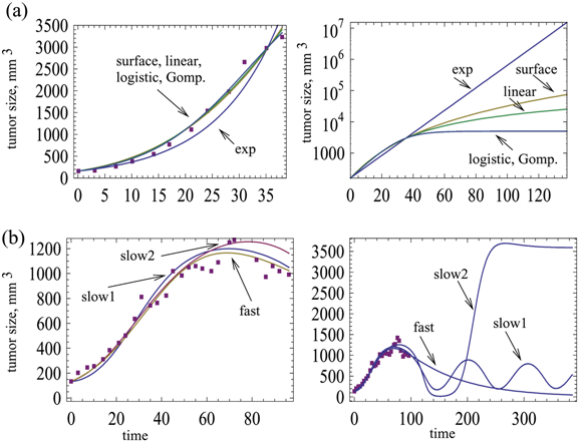
(a) Data on the growth of A549 human lung cancer nude mouse xenografts in the absence of the virus [28]. Different tumor growth models were fitted, see Table 2. The parameter values and the root mean square values are summarized in the Supporting Information S1. The graph on the right plots the predicted long-term growth curves. (b) Growth dynamics in the presence of the wild-type virus Ad309, which was injected into an established tumor. Both a slow model and a fast model were fitted. For the slow model, *G = x/(x y^1/3^+ε)*. For the fast model, *G = x/(x+y+ε)*. Tumor growth was assumed to be logistic, *F = 1−(x+y)/W*. For the slow model, different parameter combinations are shown that fit the data to a similar degree (slow1, slow2). The graph on the right shows the predicted long term dynamics. Parameter values and root mean square values are given in the Supporting Information S1.

**Figure 7:**
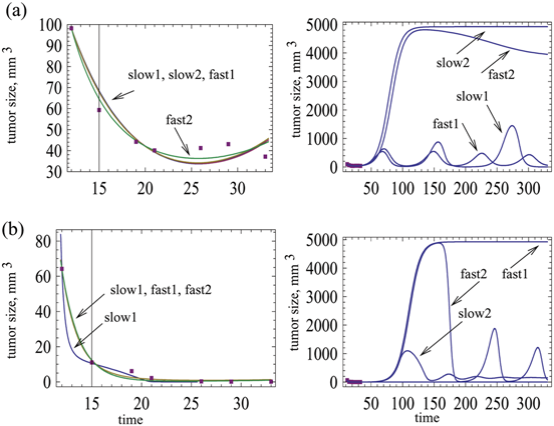
(a) Data on the growth of A549 human lung cancer nude mouse xenografts in the presence of the wild-type virus Ad309, assuming that infected and uninfected cells were mixed before the tumor cells were injected into the mouse [28]. A slow and a fast model were fitted. For each model, different parameter combinations were found that fit the model comparably (slow1, slow2, fast1, fast2).The graph on the right side shows the predicted long term dynamics for the different models and parameter combinations. (b) Infection with the mutant As337 virus, where again the infected and uninfected cells were mixed before the tumor was injected into the mice. Again, a slow and a fast model were fitted, and with each model different parameter combinations were found that provided a comparable fit to the data. As before, the graph on the right hand side shows the predicted long-term dynamics. For the slow model, *G = x/(x y^1/3^+ε)*. For the fast model, *G = x/(x+y+ε)*. Tumor growth was assumed to be logistic, *F = 1−(x+y)/W*. Parameter values and root mean square values are given in the Supporting Information S1.

Here we fit our models to previously published experimental data and discuss implications for model validation, model selection, and further experimental work. We examined data published by [28]. This study considered A549 human lung cancer nude mouse xenografts, and infected them with the wild-type adenovirus Ad309 and a mutant virus Ad337 (characterized by a deletion in the E1b-19kD gene). The resulting dynamics were investigated under two conditions. (i) Under the first condition, the cancer cells were used to establish subcutaneous tumors in the mice. When the tumors reached a certain size, the virus was injected into the tumor. (ii) In a second scenario, infected cells were first mixed with uninfected cells, and the mixture was injected into the mice. The first scenario corresponds to spatially more restricted virus growth, while in the second scenario there is a higher degree of mixing between infected and uninfected tumor cells due to the experimental protocol. For both scenarios, we fitted models that differ in the infection term *G* and the tumor growth term *F*. We performed non-linear least squares regression, using standard software. The exact models that were used are provided in Figures 6 and 7. The parameter estimates obtained for all fits are tabulated in the Supporting Information S1.

We first fitted the control tumor growth in the absence of the virus (Figure 6(a)). Both exponential growth and saturated growth models (logistic, gompertzian, surface and linear, see table 2) were applied. The saturated growth models fit the data better than exponential growth. All saturated growth models fit the data well, the logistic growth yielding the lowest (by a small margin) root mean square (RMS) error. For convenience, we chose logistic tumor growth as the basis for analyzing the effect of virus infection.

First, consider experimental condition (i), where the tumor was allowed to grow in the mice before the virus was inoculated. Only the wild-type virus Ad309 is considered. In this experiment, tumor growth was significantly reduced by the virus. However, tumor size reached a plateau by day 50, despite the persistence of the virus, leading to the conclusion that the virus failed to eradicate the tumor cell population. For fitting purposes we considered one fast and one slow virus spread term, the first and the third in table 1. Figure 6(b) shows that both a fast and a slow virus growth model can fit the data. However, extrapolating beyond the experimental time frame, very different long term outcomes are observed. The fast model predicts that the tumor remains at relatively low levels, controlled by the persisting virus infection. With the slow model, we show two parameter combinations which both fit the data well, but which are characterized by different long term outcomes. For one parameter combination (slow 1), damped oscillations are observed that lead to persistence of both the tumor and the virus at relatively low levels. For the second parameter combination (slow 2), the tumor cell population escapes control and grows to high levels. The virus population persists at low and ineffective levels (not shown). Therefore, not only do different models predict different long term dynamics; within one model, different parameter combinations that describe the data equally well can give rise to different predictions regarding the long-term dynamics. Our discussion of this and other results is postponed until the end of this section.

Next consider experimental condition (ii), in which infected cells were mixed with uninfected cells at a ratio of 1∶1000 before the tumor was injected into the mice. In this case, the viruses were generally more effective. Tumor growth was prevented, and the number of tumor cells declined to low levels. Figure 7 fits a slow and a fast model to data that document infection with the wild type virus Ad309 (Figure 7a) and the mutant virus Ad337 (Figure 7b). Consider the wild type As309 virus first (Figure 7a). All models fit the data well. Again, the predictions about the long-term dynamics vary, not only between models, but between different parameter combinations of the same model. Two qualitatively different outcomes are depicted in Figure 7a. On the one hand, the cancer can grow out of control following the initial reduction in the number of cancer cells. On the other hand, the virus maintains control of the cancer, which persists but is suppressed to relatively low levels. Thus, the encouraging but limited trend shown by the data cannot be used to conclude efficient virus-mediated tumor control. Longer experimental studies are needed in order gain insights into the eventual outcome of treatment, and to differentiate between the various model predictions. Figure 7b shows the same analysis for the mutant virus Ad337. As before, the slow and fast model can both fit the data well, and within one model, different parameter combinations are possible. The long-term dynamics show different outcomes, depending on the model and the parameter combinations. They include long term virus-mediated cancer control, as well as uncontrolled cancer growth. In the context of the experimental data, however, these long-term dynamics will not be observed, as the cancers regressed completely in the experiments. During the initial decline of the cancer cell population in the model, the number of cells drops to such low levels that extinction is actually the likely outcome in practical terms. However, what this tells us is that if by chance the cancer cell population does not hit extinction in the experiments, it is entirely possible that the cancer cell population rebounds and grows to high levels, depending on the model and its parameters.

As mentioned above, the experiments include both a highly spatial setting where the virus was inoculated into an already established tumor, and a mixed setting where infected and uninfected cells were mixed before the tumor cells were placed into the mouse. Therefore, it can be tempting to examine whether the relative goodness of fit for the fast and slow models is different in these two situations. As explained, however, each model can fit the data with several alternative parameter combinations. There are many more solutions to the least squares regression than shown here. Therefore, it does not make sense to compare the goodness of fit for slow and fast models. For instance if the fits obtained for the slow model are slightly better than those obtained for the fast model, it is quite possible that there exists another parameter combination in the fast model that is better yet, and that has not been encountered so far. This brings us to the fundamental problem of nonlinear data fitting and model validation, which is an interesting issue in itself and will be discussed briefly here.

As with many (and perhaps most) other nonlinear models, the parameter space where the minimization of the RMS error is performed, is multidimensional and is characterized by many shallow local minima. Most standard fitting routines get “stuck” at local minima, and even more sophisticated algorithms aimed at finding the global minimum are not very useful, because the difference between the global minimum and many runner-ups is usually insignificant and cannot serve as an indicator of the “right” or “best” fit. Therefore, to attack the fitting problem, one is required to repeat the minimization procedure multiple times, either by performing an exhaustive span of the space of the initial guesses, or by implementing a Monte-Carlo method. The statistics of the outcomes are then analyzed in the hope to find clusters of good fits, which are then assumed to be indicative of the solution of interest.

The unfortunate part is that most of the times, these sophisticated statistical techniques are not very useful because the data sets that the fitting is applied to are simply too sparse, and they probably do not contain enough information to distinguish between models. Some of the deficiencies of experimental data sets are (i) an insufficient number of time-points, (ii) a very large experimental error at each time point, due to the experimental difficulties as well as a small sample size, and (iii) the long-term dynamics is often not captured due to the time-constraints of the experiment. In other words, no sophisticated statistical data manipulation can help distinguish between models if the data set is too sparse, short and contains large scatter.

What can we conclude from these considerations and our own attempts to validate the models based on published experimental data? The good news is that at least some of the models contain parameter combinations which describe the existing data reasonably well. The bad news is that model validation/rejection was not possible in the particular system that we used. If data were collected over longer periods of time, and with a larger sample size, then the number of parameter combinations that can fit the data would be significantly reduced, and allow for more meaningful model comparison.

## Discussion

In this paper we presented the first modeling approach that tries to analyze the dynamics of oncolytic viruses in a general setting, going beyond particular models in which results can easily depend on mathematical terms chosen. Previous approaches to modeling oncolytic virus dynamics, and virus dynamics in general, have been based on particular models that include uncertain and unrealistic assumptions. The most striking is the assumption about the infection term, which usually assumes perfect mixing of populations, and which is certainly violated in any biologically realistic setting.

Our method can be considered a hybrid between such space-free, mass-action approaches, and much more complex methods involving spatial network ideas, e.g. [29]–[36]. The former approach fails to capture spatial and geometric constraints which play an important role in infection spread. The latter approach is only analytically tractable to a certain degree; also, it usually relies on a particular, given, set of rules that govern the infection spread. Our investigation aims to capture general trends that arise from different assumptions on the infection mechanism. It combines the analytical tractability of simple dynamical systems with a more realistic modeling of infection spread.

We found that based on the infection term, we can divide models into two categories with fundamentally different behavior. In one group, virus growth is relatively fast because the infected cells are dispersed among the uninfected cells rather than being clustered together. In this case most infected cells contribute to virus spread. In these models, there is a clear viral replication rate threshold beyond which the number of cancer cells drops to levels of the order of one or less, corresponding to extinction in practical terms. Under this parameter region, this is the only outcome in this class of model. In the other category, infected cells are assumed to be clustered together to some degree in a mass, which might be realistic for solid tumors. In this case, only the infected cells located at the surface of the cluster contribute to virus spread because they are in the vicinity of uninfected cells. The infected cells located in the center of the cluster are surrounded only by other infected cells and therefore do not contribute to virus replication. The larger the number of infected cells, the smaller the proportion of cells that can pass on the virus. In this scenario, virus therapy is more difficult. If tumor growth saturates only at relatively large sizes or does not saturate, then even in the parameter regions where the dynamics can converge to tumor control or eradication, there can be the possibility that the cancer can outrun the virus if the number of cancer cells lies above a threshold at the start of virus therapy. This is because of the existence of the saddle node equilibrium which ensures dependence of the outcome on initial conditions. This might be problematic in clinical settings, because there is only a relatively small window between the size at which the tumor becomes detectable (about *10^10^* cells) and the size at which it can induce mortality (around *10^13^* cells).Tumor growth saturation at lower levels introduces a parameter region in which only the tumor control outcome is possible. A further reduction in the number of tumor cells at which growth saturation occurs can abolish the existence of the saddle node equilibrium altogether. In this case, the only outcome is tumor control. This result makes intuitive sense: earlier saturation of tumor growth slows down the cancer, and makes it easier for the virus to gain the upper hand. It also means that if the tumor is found early, it might be possible to slow down tumor growth by means of more conventional drug therapy, enabling the virus to control the cancer and to prevent runaway growth. There is indication in clinical data that a combination of chemotherapy and oncolytic virus therapy leads to better results than either approach alone [37].

Another important finding of our study is that the basic results regarding the outcome of oncolytic virus therapy do not depend on the particular tumor growth terms used in the model. The exact kinetics of tumor growth are still poorly understood and a source of uncertainty. We examined straight exponential growth, as well as a number of more realistic options, including saturated but continued growth at high numbers of cancer cells, as well as cessation of growth as the number of tumor cells approaches an upper limit. While there are minor differences (such as the existence of a stable equilibrium at large tumor sizes vs continues slow growth), the properties of the tumor control equilibrium are largely independent from the exact way in which tumor growth is modeled.

Throughout this paper we discussed the ability of the virus to eradicate the tumor in the context of our mathematical model that aims to describe oncolytic virus growth in relatively simple settings. It is important to point out that even simple scenarios could be characterized by complicating conditions which are not captured in the model and which make actual tumor extinction difficult to achieve. For example, tumor cells might become resistant to the virus by for example down-regulating the receptor required for viral entry [38]. Related to this, cells could temporarily become resistant to virus-induced effects depending on the stage of the cell cycle [39]. Such effects can be easily incorporated into our framework, if data suggest that they play a role in determining the dynamics of oncolytic virus growth.

The framework presented here aims to bring us closer towards predictive computational models of oncolytic virus replication in vitro. Both additional computational and experimental work will be necessary to advance this framework. On the theoretical side, it will be important to also explore spatially explicit and stochastic models. The ordinary differential equations are desirable because they can be applied to experimental data in a relatively straightforward way. At the same time, however, spatial aspects of population growth can only be captured in a phenomenological way. Hence, it will be important to consider a spatially explicit model and to compare its properties to the results obtained here. On the empirical side, it will be important to run experiments that document the growth of specific oncolytic viruses e.g. in a culture of specific tumor cells. These data can be fitted to the various models explored here to determine which model describes the data best and which models can be rejected. This can be done in a variety of setting: a culture where cells and viruses can mix well; a 2D tissue culture which imposes a degree of spatial constraints; and a 3D tissue culture which can impose further spatial constraints. Different models will apply to these different scenarios. This will allow us to test the theoretical notions presented here, and to obtain a set of models that are predictive for the relevant scenarios.

Of course, for clinical relevance, oncolytic virus replication needs to be considered in the context of more complex settings. Most importantly, the virus is immunogenic, and immune responses can inhibit the spread of the virus and can even drive it extinct. Such components will have to be incorporated into a mathematical model that describes the replication of an oncolytic virus in vivo. However, before we have obtained a solid understanding of the principles that govern the dynamics of oncolytic viruses in simpler settings, it is unlikely that modeling can contribute much to understanding the more complicated in vivo scenario. The modeling framework discussed here provides a basis to incorporate increasing amounts of biological complexity in the future, and thus to gradually improve our understanding of the key factors that determine the outcome of oncolytic virus therapy.

## Materials and Methods

The results described in this paper are based on the analysis of ordinary differential equations. Extensive mathematical details are provided in the Supporting Information S1.

## Supporting Information

Supporting Information S1 (0.11 MB PDF)
