# Supplementary material for: Towards Predictive Computational Models of Oncolytic Virus Therapy: Basis for Experimental Validation and Model Selection
Source: PLoS One. 2009 Jan 30;4(1):e4271. doi: 10.1371/journal.pone.0004271 (PMC2629569; doi:10.1371/journal.pone.0004271)
Supplement: Supporting Information S1 — (0.11 MB PDF) [file pone.0004271.s001.pdf]

Towards predictive computational models of oncolytic virus therapy: basis for experimental validation and model selection

Dominik Wodarz and Natalia L. Komarova

Supplementary Materials

1 Axiomatic model construction

We assume that the cancer growth term satisfies the following biological requirements:

1. The obvious requirement of positivity: $F(x, y) \geq 0$ for all $x, y \geq 0$, and continuity.
2. At the beginning, the growth is exponential: $\lim_{z \to 0} F(z) = 1$.[1]
3. The growth slows down as the number of cells increases: $dF(z)/dz \leq 0$.
4. A symmetry requirement: $F(x, y) = F(x+y)$: the growth is controlled by infected and uninfected cells equally; note however that this requirement is not essential for our analysis.

We assume that the virus spread term ($\beta y G(x, y)$) satisfies the following biological requirements:

1. The obvious requirement of positivity: $G(x, y) \geq 0$ for all $x, y \geq 0$, and continuity.
2. For small values of x and y, the growth should be exponential to reflect perfect mixing:
$$\lim_{x,y \to 0} G(x, y)/x = 1.$$
3. The growth term must monotonically increase with x and y:
$$\frac{\partial (yG(x, y))}{\partial x} \geq 0, \quad \frac{\partial (yG(x, y))}{\partial y} \geq 0.$$
4. The growth *rate*, $G(x, y)/x$, must slow down with x and y:
$$\frac{\partial (G(x, y)/x)}{\partial x} \leq 0, \quad \frac{\partial (G(x, y)/x)}{\partial y} \leq 0.$$

[1] This requirement fixes the scaling of the time-variable. In general, if the initial growth-rate $\lim_{z \to 0} F(z) = r$, we scale time $t' = tr$, and also use $a' = a/r$ and $\beta' = \beta/r$. The primes are dropped for convenience.

5. The growth has to be saturated in both x and y, such that

$$\lim_{x\to\infty} yG(x,y) = H_x(y), \quad 0 < H_x(y) < \infty,$$

where $H_x(y)$ is a function of y independent of x. Similarly, with y.

6. For large values of x, the growth term cannot be positive in the limit of small y, that is,

$$\lim_{y\to 0} H_x(y) = 0.$$

Similarly, with x.

7. For large values of x and y the spread cannot stop completely:

$$\lim_{x,y\to\infty} yG(x,y) > 0.$$

Note that this expression could be infinite.

The analyses below rely on these properties.

2 The number of equilibria

The fixed points of the virus-cancer system (equations (1-2) of the main text) are given by $(0,0)$ and all the solutions of the equations

$$xF(x+y) = ay, \tag{1}$$

$$G(x,y) = \frac{a}{\beta}. \tag{2}$$

The trivial point $(0,0)$ has eigenvalues $F(0)$ and $-a$ and is thus a saddle. The number of solutions of equations (1-2) depends on the particular shapes of the functions F and G. In order to find the nontrivial equilibria, we solve equation (1) to find $y(x)$, and then substitute it into equation (2). The equilibria are thus defined by the roots of equation

$$\beta G(x, y(x)) = a. \tag{3}$$

From equation (1) we can see that $y(0) = 0$. We know from assumption (2) on the function G that $G(0,0) = 0$. The next step is to study the limiting behavior of $G(x, y(x))$ for large values of x. For that we need to know the behavior of $y(x)$ for large x. We have from equation (1):

$$\lim_{x\to\infty} y(x) = \lim_{x\to\infty} xF(x)/a.$$

There are three cases. (i) For a linear type growth, we have $\lim_{x\to\infty} xF(x) = c_0$, a nonzero constant. In this case, $\lim_{x\to\infty} y(x) = c_0/a$, with $0 < c_0 < \infty$. (ii) For any growth F which is superlinear but slower than exponential, we have $\lim_{x\to\infty} y(x) = \infty$, but $\lim_{x\to\infty} y/x = \lim_{x\to\infty} F(x+y)/a = 0$, that is,

y increases slower than x. (iii) Finally, for exponential growth, $F = 1$ and $y(x) = x/a$, such that $y(x) \sim x$ for large values of x.

From the biological assumptions on the function $G(x, y)$ listed above, it follows that for any of the possible dependencies $y(x)$, the function $G(x, y(x))$ approaches a finite limiting value as $x \to \infty$, and this value can be zero or nonzero. We use the exponential case $F = 1$ and $y_{exp}(x) = x/a$ to separate all functions G into two classes. If $\lim_{x\to\infty} G(x, x/a) = 0$, then we will consider the virus spread to be *slow*, and if $\lim_{x\to\infty} G(x, x/a) = G^{\infty}_{exp} > 0$, with $G^{\infty}_{exp} < \infty$, we will regard this as *fast* spread. Note that for all laws of cancer growth slower than exponential, we have $G(x, y(x)) \geq G(x, y_{exp}(x))$. This is because $y(x) \leq y_{exp}(x)$, and G is a decreasing function of y.

2.1 Fast virus spread

In this case, the function $G_{exp}(x) \equiv G(x, y_{exp}(x))$ is either a monotonically increasing function (figure 1(a) of the main text), or it can attain one or more local extrema before converging to its nonzero horizontal asymptote.

For a monotonically increasing G_{exp}, low values of β correspond to zero roots in equation (2), which means that the cancer growth will continue indefinitely (figure 1(a) of the main text). As β crosses a critical value defined by a/G^{∞}_{exp}, there is one root. The value of x at this root drops as β increases (this is due to the convergence of G_{exp} to an asymptote, G^{∞}_{exp}). For large values of β, the value of x at the intersection tends to zero.

If the function G_{exp} has an absolute maximum at point x_{exp}, then an initial increase of β above a/c_{max} with $c_{max} = G_{exp}(x_{exp}, y(x_{exp}))$ results in the appearance of two roots. Additional local extrema will result in an appearance and disappearance of pairs of roots. However, as β increases through the second threshold given by a/c_2, only one (the lowest) root remains. The value of c_2 is given by the lower of the values $\{G^{\infty}_{exp}, c_{min}\}$, where c_{min} is the value at the lowest local minimum, if it exists.

In both cases, for sufficiently large values of β, there will be only one root in equation (2). Introducing other cancer growth laws can increase the limiting value of G thus decreasing the value of β_c. In the case of a monotonically increasing G_{exp}, there will be no qualitative change. If G_{exp} is one- or multiple-humped, the hump(s) may disappear. Whether this qualitative change happens depends on the relative size of the two spacial scales involved. The first scale is defined by the location of the maxima of G_{exp} and is related to the virus spread scale, s_v. The second scale is given by the size, s_t, at which cancer growth law starts to deviate from exponential. Once $s_t \sim s_v$, the limiting value of G becomes sufficiently large such that the "hump" disappears.

It is useful to investigate the value of x at the equilibrium as a function of β, for different values of s_t. Suppose that the graph of $G(x, x/a)$ is a monotonically growing function of x which approaches a limiting value, G^{∞}_{exp}. Suppose that the cancer growth slows down around the scales near s_t. So near $x \sim s_t$, the function $G(x, y(x))$ deviates from the horizontal asymptote, G^{∞}_{exp}, and starts growing toward a different, and higher horizontal asymptote, which we will call

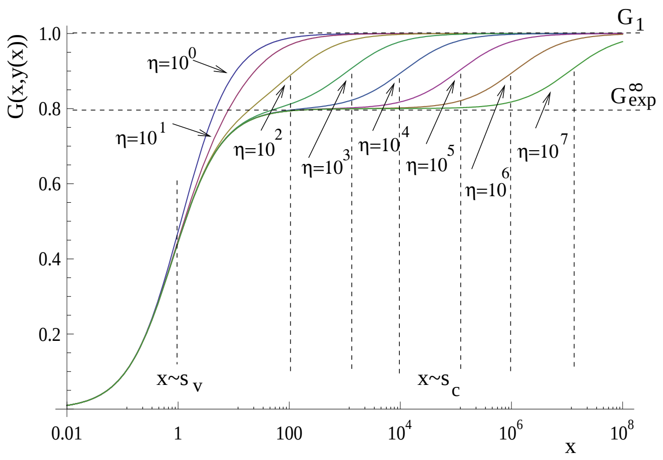

Figure 1: The dependence of the equilibrium on β and s_t. We use the model with $F = \eta/(\eta + x + y)$ (thus, s_t is defined by η) and $G = x/(x + y + \epsilon)$. The function $G(x, y(x))$ is plotted vs x for different values of η. The dashed vertical lines indicate the scales of interest: the leftmost such line corresponds to $x \sim s_v$, and the rest of the lines to $x \sim s_t$ for different values of η. The other parameters are: $a = 4$, $\epsilon = 1$.

$G_1 > G^{\infty}_{exp}$ (see figure 1 for a particular example). The phase diagram as β increases is as follows. For $\beta < a/G_1$, there are no roots. As β crosses the first threshold, a/G_1, one root appears. The value of x at this equilibrium decays rapidly from infinity to values around s_t, as β grows (because of the fact that G_1 is a horizontal asymptote). Then as β grows through its second threshold, a/G^{∞}_{exp}, the value of x at the equilibrium drops from s_t to values of order 1. The second transition is sharp if the following is satisfied: $s_t \gg x_1$, where x_1 is the value of x such that $|G(x_1, y(x_1)) - G^{\infty}_{exp}| = |G_1 - G^{\infty}_{exp}|$. In other words, x_1 is the value of x where the function $G(x, y(x))$ comes near its first horizontal asymptote ("near" means that it is at least as close to G^{∞}_{exp}, as G^{∞}_{exp} is to G_1). If $s_t \gg x_1$, then the function G has a significant interval in x where it comes near the value G^{∞}_{exp}, before it deviates from it to start growing toward G_1. This guarantees a threshold effect.

We conclude that for all cancer growth laws and for all functions G corresponding to a fast virus spread, increasing β beyond a threshold leads to the existence of only one equilibrium, whose value correlates negatively with the viral replication rate, β. For large enough s_t, there is a "threshold" effect, such that the size at equilibrium decreases very sharply as β approaches a defined value.

2.2 Slow virus spread

In this case, the function $G_{exp}(x) \equiv G(x, y_{exp}(x))$ is a one- or a multiple-humped function, which approaches zero for large x (figure 1(b) of the main text).

In the case of an exponential growth, the bifurcation diagram looks as fol-

lows. As before, small values of β correspond to no equilibria (zero roots in equation (2)). As we increase β, a pair of roots appears after the threshold given by a/c_{max}, where $c_{max} = G(x_{exp}, y(x_{exp}))$, the maximum value of G_{exp}. As β increases, other roots may appear and disappear in pairs. Since $G_{exp}(0) = 0$ and the function G_{exp} has zero as its horizontal asymptote, there will be two equilibria for all values of β larger than a/c_{min}, where c_{min} is the value of G at its lowest local minimum in the case that such a minimum exists; $c_{min} = c_{max}$ otherwise.

Let us next consider how non-exponential laws of cancer growth modify this picture. In some cases, a slower-than-exponential growth term, F, will lead to the horizontal asymptote of $G(x, y(x))$ becoming nonzero. In general whether this happens depends on the functional forms of both G and F. In the case of linear growth, $y(x) \equiv y_{lin}(x)$ converges to a nonzero constant, c_1, and we have $\lim_{x\to\infty} G_{lin}(x) = \lim_{x\to\infty} G(x, c_1) = G_x(c_1) = c_2 < \infty$, which is a nonzero constant. Depending on the value of s_t, G_{lin} can be a one- or a multiple-humped function, or (for s_t similar or smaller than x_{exp}) it is a monotonically increasing function of x. In either of these cases, there exists a finite value of β such that for all values of β larger than this value, there is only one root in equation (2). For growths faster than linear but slower than exponential, we have $y \to \infty$ as x grows, but $y = o(x)$, i.e. it grows slower than x. In some cases the function G will retain a zero asymptote (e.g. in the case where $G = x/(x+1)/(y+1)$ and a surface growth law for F). In other cases it will acquire a nonzero limit (e.g. with $G = x/(x + 1 + \sqrt{x}(y+1))$ and a surface growth law for F). In the latter case we can say that the surface cancer growth is sufficiently slow to warrant successful treatment given the particular mode of viral spread.

2.3 Finite tumor size

Here we consider a growth term which becomes zero for a finite value of $x + y$. The growth starts off exponential ($F(0) = 1$) and at some size, s_t, it slows down (we do not exclude the possibility that $s_t \sim 1$, that is, the growth becomes slower than exponential right away). Then there exists another characteristic size, $W \gg s_t$ such that the growth slows down further and stops. We define W such that $F(W) = 0$. Note that if $s_t \sim W$ then there is no need to introduce the two scales, s_t and W. Therefore, the assumption $s_t \ll W$ must hold.

The previous analysis holds on the scales intermediate between s_t and W, such that $s_t \ll x \ll W$. In particular, for values $x \ll W$, the shape of the curve $G(x, y(x))$ is similar to that obtained for the corresponding unlimited growth. As x grows far beyond s_t and approaches W, the function G approaches $G(W, 0)$. If, for the unbounded growth, the limiting value of the G function is c_2, we have in general $G(W, 0) \geq c_2$, and the curve G takes an upward turn in the vicinity of $x = W$. This means that equation (2) acquires an additional root corresponding to the cancer growing to its carrying capacity, W. In the systems with unrestricted growth this was equivalent to an unlimited growth of the cell population.

It is useful to note the following: in systems with a limited size, the function

$G(x, y(x))$ is always bounded away from zero. Therefore, strictly speaking, we can always find a threshold value β_t such that for $\beta > \beta_t$, only one root is present. However, if $W \gg s_v$, such values of β are very large compared to β_c, and in most cases are probably not achievable.

3 Stability

Let us suppose that (x_0, y_0) with $x_0 \geq 0$ and $y_0 \geq 0$ is a solution of system (1-2), and consider the stability of the corresponding equilibrium. The Jacobian of the system can be written as a 2×2 matrix, $\{m_{ij}\}$, with

$$m_{11} = F + x_0 F' - \beta y_0 G_x, \quad m_{12} = x_0 F' - \beta(G + y_0 G_y),$$
$$m_{21} = \beta y_0 G_x, \quad m_{22} = \beta y_0 G_y,$$

where the functions F and G and their derivatives are evaluated at the point (x_0, y_0): $G_x = \partial G/\partial x|_{x=x_0, y=y_0}$, and similarly with G_y and F'. The equilibrium is stable if the following two conditions hold:

$$m_{11} + m_{22} < 0, \tag{4}$$
$$m_{11} m_{22} - m_{21} m_{12} \geq 0. \tag{5}$$

3.1 Saddle points

Condition (5) is equivalent to the positivity of the derivative of G in the direction defined by the implicit relation $ya = xF(x + y)$, equation (1). Differentiating it, we get: $ady = Fdx + xF'(dx + dy)$. The directional derivative is equal to $(G_x dx + G_y dy) = [G_x(a - F' x_0) + G_y(F + x_0 F')]/(a - F' x_0)$. The denominator is positive, so this expression has the same sign as the left hand side of condition (5).

The equilibria are defined by the roots of equation (3). Since $G(0, 0) = 0$, all the odd roots of equation (3) will correspond to a positive, and the even ones to a negative slope of the left hand side of equation (3). This means that all even equilibria are saddles. This is because in such cases the directional derivative is negative, condition (5) is violated, and therefore there are two real eigenvalues of opposite signs. On the other hand, an odd root can be either a sink, a source or a spiral (stable or unstable). This is because for such a root, condition (5) is always satisfied, so that we could either have complex eigenvalues, or real roots of the same sign (positive or negative).

In the presence of a saddle, an infinite outcome (corresponding to an unchecked cancer growth) is possible. For large values of x, we have

$$\dot{x} = xF^\infty - \beta y G^\infty(y), \tag{6}$$
$$\dot{y} = y(\beta G^\infty(y) - a), \tag{7}$$

where $\lim_{x\to\infty} G(x, y) = G^\infty(y)$ and $\lim_{x\to\infty} F(x, y) = F^\infty$. The growth of y becomes negative as y increases if $\lim_{y\to\infty} G^\infty(y) = 0$, which suggests that y

settles to a finite value which makes the right hand side of equation (7) zero, such that the outcome $(\infty, const)$ is observed. If $\lim_{y\to\infty} G^{\infty}(y) = const > 0$, then for large enough values of β we can have an outcome of the form (∞, ∞).

3.2 Properties of the internal equilibrium

Let us first show that for large values of β, there will be an equilibrium, (x_0, y_0), such that $\lim_{\beta\to\infty} x_0 = 0$ and $\lim_{\beta\to\infty} y_0 = 0$. We call this equilibrium the "internal equilibrium". Its existence follows from equation (3) and the properties of the function G. We know that $y(0) = 0$, and also that $G(0, 0) = 0$. It is also clear that there is an interval of x, $[0, \xi]$, where G is a growing function. Therefore, by continuity, for all $\beta \geq a/G(\xi, y(\xi))$, there will be a solution of equation (3). From monotonicity of the function G, the value of x at the intersection with a/β decays with β. From equation (1) it follows that there is an interval of x, $[0, \xi_1]$, where y is a growing function of x. Therefore, we conclude that for large enough β, there is an equilibrium whose x and y values decay with β and approach 0 in the limit $\beta \to \infty$.

Let us evaluate the left hand sides of inequalities (4) and (5) for small values of x_0 and y_0. First, we express β from equation (2): $\beta = a/G(x_0, y_0)$. Then we approximate the curve $y(x)$ by its Taylor series for small values of x_0:

$$y_0 = Fx_0/a+(a+F)F'(x_0/a)^2+(a+F)((F')^2+1/2(a+F)F'')(x_0/a)^3+O[(x_0/a)^4], \tag{8}$$

where the function F and its derivatives are evaluated at 0. This expression follows from expanding both sides of equation (1) in Taylor series in terms of x_0 and y_0, solving for y_0 and using a Taylor expansion of this expression. Now, let us multiply the left hand side of inequality (4) by $G(x_0, y_0)$, and use expression (8). Expanding in terms of small x_0, we obtain:

$$\begin{aligned} &G(x_0, y_0)(m_{11} + m_{22}) = (F'G_x + G_{xy} - G_{xx}/2)x_0^2 \\ &+\frac{1}{a}\left((a+1)F''G_x + (a+2)F'G_{xy} + G_{xyy} + \frac{1}{2}((a-1)G_{xxy} - F'G_{xx}) - \frac{1}{3}aG_{xxx}\right)x_0^3 \\ &+O([x_0]^4). \end{aligned} \tag{9}$$

Here the functions F and G and their derivatives[2] are evaluated at zero. To derive the above expression we also used the fact that the function G and its y-derivatives are equal to zero if $x = 0$, and $F(0) = 1$.

Next, we evaluate the left hand side of inequality (5) in the same manner:

$$G(x_0, y_0)(m_{11}m_{22} - m_{21}m_{12}) = aG_x x_0 + (aF'G_x + 2G_{xy} + aG_{xx})x_0^2 + O([x_0]^3).$$

We can see that the expression above is always positive, so condition (5) is satisfied for large enough values of β. This means that the two eigenvalues (if real) have the same sign. Condition (4) however is not necessarily satisfied,

[2]Here we assume that the functions F and G are differentiable at zero. Non-differentiable functions are handled similarly by using generalized expansions.

Growth law	Fitted formula	RMS error
Exponential	$F = 0.083$	271.6
Logistic	$F = 0.11(1 - x/(4.9 \times 10^3))$	96.2
Surface	$F = 0.81/(x + 134.6)^{1/3}$	137.0
Linear	$F = 265.0/(x + 22 \times 10^3)$	119.6
Gompertz	$F = 2.80x \log[1.32 \times 10^5/(x + 1.27 \times 10^5)]$	96.6

Table 1: Fitted growth functions for figure 6, and their root mean square errors.

Figure	Model	ϵ	β	a	f_0	RMS error
6(b)	slow1	790.6	2.06	0.274	0.26	96.5
6(b)	slow2	537.3	1.4	0.169	0.198	104.4
6(b)	fast	40.0	0.152	0.011	0.40	89.4
7(a)	slow1	755.0	2.109	0.17	0.82	6.00
7(a)	slow2	6×10^{-5}	0.189	0.195	0.79	5.76
7(a)	fast1	92.0	0.50	0.2	0.77	5.72
7(a)	fast2	2×10^{-7}	0.71	0.69	0.34	4.18
7(b)	slow1	0.1	3.44	4.03	0.53	1.23
7(b)	slow2	410	3.37	0.56	0.996	1.90
7(b)	fast1	0.1	1.08	1.11	0.60	1.56
7(b)	fast2	59.8	0.92	0.56	0.995	1.90

Table 2: Fitted model parameters for figures 6(a) and 7(a),(b).

as follows from expression (9). The expansion can be positive or negative, depending on the particular properties of the functions F and G.

Next, we would like to investigate whether the eigenvalues are real or complex. For the eigenvalues to have an imaginary part, the following condition has to be satisfied:

$$(m_{11} - m_{22}) + 4m_{12}m_{21} < 0. \qquad (10)$$

Performing a Taylor expansion of the above expression for small values of x_0 and y_0 at internal equilibrium, we obtain:

$$G(x_0, y_0)((m_{11}-m_{22})+4m_{12}m_{21}) = -4aG_x^2x_0^2-2G_x(2aF'G_x+6G_{xy}+3aG_{xx})x_0^3+O([x_0]^4).$$

We can see that this quantity is always negative. Therefore, we conclude that the internal equilibrium has complex eigenvalues for sufficiently large values of β.

4 Fitting experimental data

The experimental data of [1], figures 1 and 5, was used to fit with the models. The fitting was performed by using Mathematica, which allows to perform non-linear least squares fitting procedure on solutions of ODEs defined on a set of

unknown parameters. The root mean square (RMS) error was calculated as

$$\sqrt{\frac{1}{n}\sum_{i=1}^{n}(f(t_i)-x_i)^2}.$$

Here n is the number of experimental data points in the time-series, and (t_i, x_i) are the coordinates of the experimental points, where t_i values correspond to time and x_i correspond to the cancer size, in mm^4. The fitted function obtained as a solution of ODEs is denoted as $f(.)$.

First we used various growth laws to fit with the data of [1] (figure 1), where the growth of uninfected cancer cells was measured. The results are presented in figure 6(a) of the main text; the best-fitting parameters are listed in Table 1. We then used the Logistic growth model to fit the data pertaining to cancer-virus interactions.

The virus growth terms used were $G(x, y) = x/(x+y+\epsilon)$ for the fast spread model and $G = x/(xy^{1/3} + \epsilon)$ for the slow spread model (both contain only one parameter). Thus, there are 4 unknown parameters to fit: ϵ, β, a, and f_0, the initial fraction of infected cells at the time when the first measurement took place. Some examples of well-fitting parameter sets are presented in table 2.

References

[1] Harrison, D., Sauthoff, H., Heitner, S., Jagirdar, J., Rom, W. N., Hay, J. G. 2001. Wild-type adenovirus decreases tumor xenograft growth, but despite viral persistence complete tumor responses are rarely achieved– deletion of the viral E1b-19-kD gene increases the viral oncolytic effect. Hum Gene Ther 12, 1323-32
